# Systematic Review of Variable Selection Bias in Species Distribution Models for *Aedes vexans* (Diptera: Culicidae)

**DOI:** 10.3390/insects16101061

**Published:** 2025-10-17

**Authors:** Peter Pothmann, Helge Kampen, Doreen Werner, Hans-Hermann Thulke

**Affiliations:** 1 Leibniz Centre for Agricultural Landscape Research, Biodiversity of Aquatic and Semiaquatic Landscape Features, 15374 Müncheberg, Germany; 2 Department of Ecological Modelling, Helmholtz-Centre for Environmental Research GmbH, 04318 Leipzig, Germany; 3 Department of Forest Science, Technische Universität Dresden, 01069 Tharandt, Germany; 4 Friedrich-Loeffler-Institut, Federal Research Institute for Animal Health, Institute of Infectology, 17493 Greifswald, Germany

**Keywords:** ecological variable classification, variable selection, model comparison, covariates

## Abstract

We conducted a systematic literature review, following PRISMA guidelines, to assess whether existing species distribution models for *Aedes vexans* reflect its known ecological requirements. This mosquito is closely associated with temporary floodwaters, making hydrological dynamics a critical factor for accurate modelling. From 28 peer-reviewed studies, we extracted 477 environmental and ecological variables and organized them into a hierarchical classification scheme with four main categories: weather, land characteristics, water characteristics, and population. We analysed patterns in variable usage and the reported importance of each variable. Our results show that flood-related variables were largely absent, despite the species’ reliance on ephemeral water bodies for reproduction. This may possibly reduce the predictive utility of existing *Aedes vexans* species distribution models. In contrast, urban-landscape variables were frequently used and often ranked as highly predictive, but such results were primarily found in studies that did not account for sampling bias.

## Simple Summary

The mosquito *Aedes vexans* is common in Europe and often appears in large numbers after floods. Because it can transmit diseases, scientists use computer models to predict where it might occur. These models rely on environmental factors such as temperature, rainfall, and land use to describe the conditions that make habitats suitable for the species. We reviewed all published studies that used such models for *Aedes vexans* to check whether they include the environmental factors that really matter for this species. We found 28 studies and analysed nearly 500 variables used to describe weather, land use, water, and human activity. Surprisingly, most models did not include information about flooding or temporary water bodies, even though these are essential for the mosquito’s reproduction. Instead, many studies focused on urban features, a choice that may reflect where people collect samples rather than where the mosquito actually lives. Our findings highlight that understanding how and where data are collected is as important as the modelling technique itself. Taking flood dynamics into account could make predictions more realistic and help to make management of potential disease risks more effective.

## 1. Introduction

Understanding species–environment relationships is central to ecological research, offering key insights into biodiversity patterns and environmental change [1,2]. Species distribution models, which predict geographic distributions, are widely used tools in this context [3]. They support diverse applications, including conservation planning for endangered species [4,5], forecasting the spread of vector-borne diseases [6] and agricultural pests [7], projecting outbreak dynamics [8,9], and identifying areas of elevated risk [10,11].

Species distribution models are based on two main types of input data: species occurrences and environmental and ecological variables. Occurrence data reflect locations where the species is present or absent and are often derived from field surveys, museum records, or citizen science initiatives. The environmental and ecological variables (hereafter referred to as variables) describe the habitat conditions at occurrence locations. Environmental factors include climate, soil, elevation, hydrology, and land cover, while ecological factors encompass species interactions such as competition, predation, mutualism, and host availability, as well as the distribution of resources like breeding or resting sites. By relating species occurrences to environmental and ecological conditions, species distribution models define environmental and ecological conditions necessary for species persistence and project potential suitable habitat across the landscape, including across unsampled areas [3].

While occurrence data are typically obtained from large databases or the literature [12,13] and methods for the identification of the best-performing model types have been extensively studied [14], selecting an appropriate set of environmental explanatory variables remains a major challenge.

Species distribution models rely on variables to characterize the ecological niche of the species [3,15,16,17,18]. The selection of variables typically begins with an initial candidate pool, with the choice of included variables informed by expert knowledge and previous studies. This pool is then often narrowed down using data-driven approaches that assess the individual contribution of each variable to overall model accuracy [19,20]. The success of this refinement depends critically on the ecological completeness of the candidate pool. If essential environmental and ecological constraints are missing, the models may suffer from bias, overlooking key habitat factors that limit species distributions [3,15,16,17,18].

We conducted a systematic review of published species distribution models for the floodplain mosquito *Aedes vexans* with two aims: first, to evaluate whether all potentially limiting habitat characteristics are adequately represented in the selection process, thereby revealing possible biases caused by missing or insufficient variables; and second, to identify the variables most frequently used and considered important across existing models. Since these variables have already proven important in diverse modelling contexts, they are also likely to perform well in future applications.

By organizing variables according to the ecological processes or conditions they represent and their reported importance, we provide a structured overview that highlights both the inclusion of key habitat characteristics and potential gaps in current modelling practices.

Understanding the biology and ecology of *Aedes vexans* is crucial for interpreting these findings. As a floodplain mosquito species, *Aedes vexans* exhibits habitat preferences and life-cycle traits that are tightly linked to dynamic water regimes.

*Aedes vexans* relies on temporary floodwaters for reproduction, which explains the importance of water-level fluctuations in shaping its habitat suitability [21,22,23]. Females preferentially oviposit near ephemeral ponds formed by river and lake flooding, where drought-resistant eggs remain viable for years until inundation triggers hatching [24,25]. Such flood events can cause rapid population expansions by creating abundant larval habitats [25]. After hatching, adult mosquitoes typically disperse away from their aquatic breeding sites to seek blood meals.

We specifically chose *Aedes vexans* for this study because it is widely distributed and represents one of the most common floodplain mosquito species, ensuring the availability of many occurrence records and previous modelling efforts. Also, the species occupies a unique ecological niche linked to ephemeral flood habitats, which makes it particularly suitable for assessing how habitat models capture ephemeral environmental dynamics. Finally, *Aedes vexans* is of great public health relevance, as it opportunistically feeds on both humans and animals [25] and serves as a competent vector for pathogens such as Zika, chikungunya, and West Nile viruses [25,26,27].

## 2. Materials and Methods

**Figure 1:**
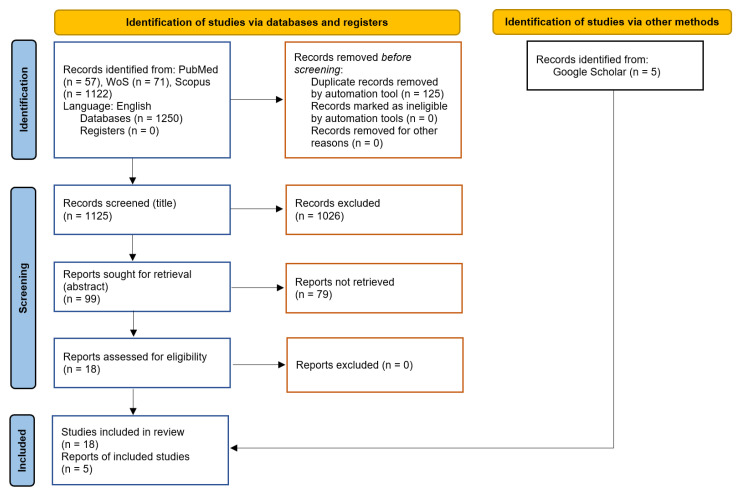
Flow diagram following the PRISMA protocol for databases, registers and other sources. Modified from [28].

We conducted a systematic literature review following PRISMA standards [28]. The PRISMA checklist and the PRISMA abstract checklist are provided in the Supplementary Materials. The search was performed in the PubMed, Scopus, and Web of Science databases on 17 April 2024. To identify a suitable search term, we first conducted an unstructured search to identify relevant studies, including those on *Aedes vexans* cited in a recent comprehensive review of mosquito species distribution models [29]. After various alternatives had been tested, the term “vexans model” was selected as the most effective. Studies were included if they developed species-specific spatial distribution models of *Aedes vexans*, without restrictions on geography or time frame. Exclusion criteria were limited to language, as only studies published in English were considered, and any studies not meeting the above inclusion criteria were excluded. Our search returned 1250 documents (Figure 1), from which we removed 125 duplicates using a tool in the Zotero software (Version 6.0.23, Vienna, VA, USA) [30]. A single reviewer screened the titles and excluded an additional 1026 documents. The remaining studies were reviewed based on their full content, resulting in the identification of 18 relevant studies. The literature-selection procedure and the subsequent data extraction were validated by a second reviewer.

To capture reports not included in major databases, two reviewers independently performed an unstructured search using Google Scholar, identifying five additional documents. This brought the total to 23 studies.

We collected all the variables originally used in the models with the aim of assessing whether key ecological requirements of *Aedes vexans* are adequately represented. To facilitate a systematic analysis, we developed a classification scheme, grouping variables into four main categories: ‘Land characteristics’, ‘Water characteristics’, ‘Population’, and ‘Weather’. Within each category, we established subcategories to provide a more detailed and precise description of the variables (Table 1). The new grouped variables act as metavariables to organize the original input variables.

Variables often referred to specific time periods, either within a single year or spanning multiple years. The time periods were collected for every model, as were the aggregation methods used to summarize the values within the periods.

Not all variables equally affect model outcomes, as their predictive importance depends on various factors [31]. Importance is typically assessed by evaluating how much model predictions change when a variable is omitted. If omitting a variable leads to substantial changes in predictions, the variable is considered significant. Variables labelled as important in the reviewed studies were recorded as such. However, differences in methods for assessing variable importance introduce some uncertainty. A detailed description of each study’s approach to evaluating importance is provided in the supplementary data [32]. To evaluate how well variables predict the distribution of *Aedes vexans*, we looked at how often each variable was used and how many studies considered it important for the model’s accuracy. The data were analysed in R (Version 4.3.3) [33]. The collected data are available in a repository for further reference [32]. This review was not registered in a recognised international registry for systematic reviews.

## 3. Results

### 3.1. Ecological Completeness of Variables

To assess the ecological completeness of species distribution models for *Aedes vexans*, we examined whether all potentially distribution-limiting variables were represented within the initial variable sets used in existing studies. Ensuring that key habitat factors are included is critical for accurately capturing the species’ ecological niche and avoiding bias in model predictions [3,15,16,17,18].

**Figure 2:**
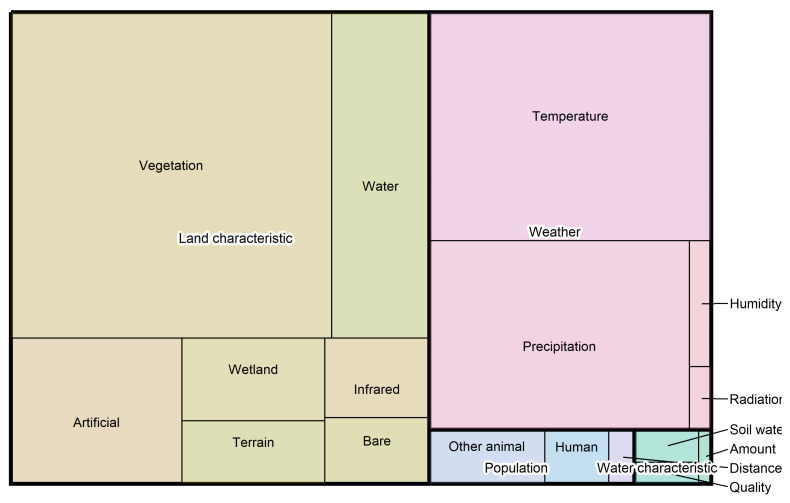
Treemap of explanatory variables used in the reviewed studies. Each rectangle represents a category of explanatory variables, with its area proportional to the number of variables used across the studies. Colour-coded boxes with white-bordered text indicate the overarching categories, while smaller boxes in matching hues represent the corresponding subcategories. If one category occupies twice the area of another, variables within that category were used twice as frequently. In total, 477 variables were included. Seven entries assigned to the subcategories ‘Missing Data’ and ‘Not specified’ were excluded from the figure and subsequent analysis.

Figure 2 provides an overview of the relative representation of different categories and subcategories among the variables used in the 28 model instances derived from the 23 selected studies on *Aedes vexans* habitat [34,35,36,37,38,39,40,41,42,43,44,45,46,47,48,49,50,51,52,53,54,55,56]. Seven variables could not be assigned to subcategories and were therefore excluded from the analysis.

The category ‘Land characteristics’ accounts for the largest share of explanatory variables, primarily as a result of the variables in the subcategory ‘Vegetation’. In addition to ‘Vegetation’, we introduced ‘Wetland’ as a separate subcategory because of the known association of *Aedes vexans* with these habitats [25]. However, ‘Wetland’ variables are used less frequently than those related to ‘Water’ or artificial surfaces (‘Artificial’). None of the variables in the ‘Water’ subcategory explicitly represent areas with fluctuating water levels, although these are a critical aspect of *Aedes vexans* biology. Such regions are represented only indirectly, for instance by ‘Wetland’ variables or elevation data classified under the ‘Terrain’ subcategory, which may serve as proxies for flood-prone areas.

The second-largest category is ‘Weather’, which primarily comprises ‘Temperature’ and ‘Precipitation’ variables, with temperature being more prevalent. Together with ‘Vegetation’, the subcategories ‘Temperature’ and ‘Precipitation’ constitute the dominant group of explanatory variables. These variables capture ecologically relevant factors identified across multiple studies and settings, suggesting they are likely to influence *Aedes vexans* distribution in future models and across different regions or conditions. For example, temperature is a known determinant of larval development and vegetation affects site selection for oviposition by female mosquitoes [21,22,23,25].

Some less frequently used variables also demonstrated strong predictive performance and may offer underexplored insights into the species’ ecological niche. Soil moisture, for instance, prevents egg desiccation and supports oviposition, yet it is rarely included despite its relevance [25]. The categories ‘Population’ and ‘Water characteristic’ were considered far less frequently.

Most variables originate from global datasets such as CORINE (20%), WORLDCLIM (20%), MODIS (13%), and GLOBCOVER (9%), despite the majority of studies being conducted at local or national scales [57,58,59,60]. Specifically, over half of the studies (58.6%) were performed at national (20.7%) or sub-national (37.9%) levels, with only a small fraction (3.5%) conducted at the global scale and 37.9% covering multiple countries without reaching a global scope. This highlights a mismatch between the spatial resolution of available environmental data and the geographic focus of most research efforts.

The high percentage of studies employing these commonly used data products reveals that projections of *Aedes vexans* distributions under future scenarios are rarely undertaken. To date, only a single study explored the species’ potential distribution under climate change, and this analysis was conducted at the global scale. No study has yet incorporated regionally downscaled climate projections to assess future distributions at the local level.

The variables used in the studies are aggregated over multiple years in 67% of cases. Nine percent are considered on an annual basis, and another nine percent are considered on a quarterly basis. The remaining variables are included in the models with greater temporal resolution.

### 3.2. Essential Variables Used in Existing Models

**Figure 3:**
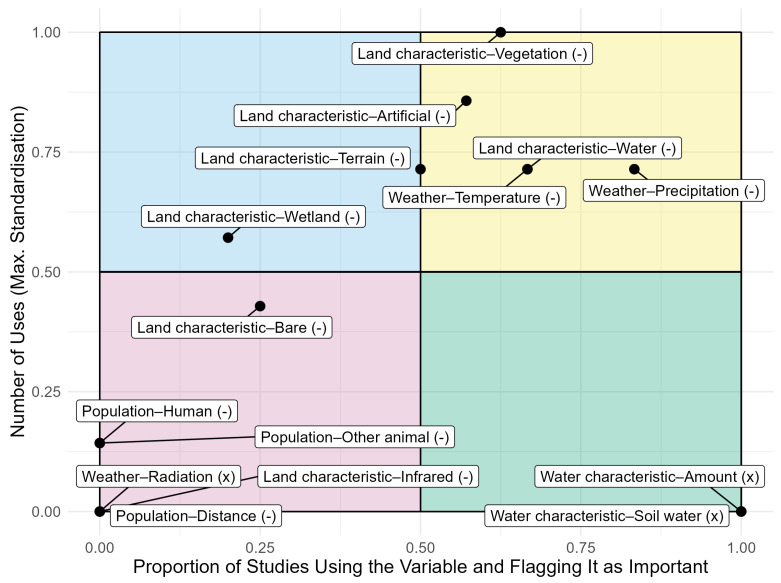
Relationship between the frequency of each explanatory variable used at the subcategory level and its perceived importance in species distribution models. The *y*-axis represents the number of times each variable was used across studies, standardised to a maximum value of 1. The *x*-axis shows the proportion of studies that not only used the variable but also identified it as important for model performance. When multiple variables from the same subcategory were included in a study, they were merged and the subcategory was considered important if any of its constituent variables significantly influenced the model outcome. The seven values of the subcategories ‘Missing data’ and ‘Not specified’ were excluded. Symbols next to variable labels indicate whether spatial sampling bias in the occurrence data was addressed in the corresponding studies. The symbol ‘-’ indicates a mixture of studies that did and did not account for sampling bias, and the symbol ‘x’ indicates none of the studies accounted for sampling bias. None of the variables was exclusively used in studies that accounted for sampling bias in occurrence data. Six studies did not use occurrence data and were excluded from this analysis.

We analysed the correspondence between the frequency of use of variables and their recognised importance in characterizing *Aedes vexans* habitat (Figure 3). The variables used in the modelling studies vary in their influence on the models’ outcomes. If a variable is removed and the accuracy of the model drops significantly, that variable is of great importance as a descriptor of the potential *Aedes vexans* distribution. This metric is known as variable importance.

Five studies had to be excluded from the analysis because they used methods which did not allow for the determination of the predictive power of variables [35,36,38,44,48]. These are primarily customised models, the use of which limits the ability to assess predictive quality between alternative model versions.

Approximately 39% of the studies reported the importance of individual variables. Among these, the most commonly used method was permutation importance or variable contribution (38%), while other approaches such as jackknife tests (25%), fitting individual models with selected variables (25%), or SHAP values (12.5%) were applied less frequently.

The yellow top-right square of Figure 3 highlights variables that are frequently used, and when applied, were often considered important for model performance. Main subcategories of variables such as ‘Vegetation’, ‘Temperature’, and ‘Precipitation’ fall into this group. In contrast, the blue top-left section includes variables that are also frequently used but rarely demonstrate a substantial influence on model results. The lower half of the figure features variables that are rarely considered. In particular, the green bottom-right box comprises variables that are seldom used but consistently flagged as important when they are included. The variables in this box are classified in the category ‘Water characteristics’.

None of the variables was exclusively used in studies that fully accounted for sampling bias in occurrence data, and therefore no ‘+’ can be found in the figure. For most variables, usage was distributed across studies that either addressed or neglected sampling bias. Notably, three variables were used exclusively in studies that did not apply any methods for sampling-bias correction.

## 4. Discussion

Species distribution models depend on the careful selection of variables to accurately represent a species’ ecological niche [3,15,16,17,18]. This study pursued two primary objectives: (1) to identify variables that are frequently used and reported as important in existing *Aedes vexans* species distribution models and are therefore likely to be relevant in future models; and (2) to detect and critically evaluate potential biases in current variable-selection practices. Our review of 23 studies and 28 model instances yielded 472 reported variables. We classified them based on their frequency of use and reported predictive importance.

Our review suggests that, with the exception of the subcategory ‘Artificial’, most variables shown in the right half of Figure 3 should be included in the initial variable selection for future species distribution models for *Aedes vexans*. In particular, variables in the upper-right quadrant were frequently used, demonstrated robust predictive performance, and represent essential dimensions of the species’ ecological niche, such as temperature, precipitation, and vegetation cover [25]. However, even these variables warrant critical ecological evaluation to ensure that key aspects of the species’ biology are not overlooked.

A prominent example of such an omission concerns the lack of variables capturing flood dynamics, despite their central importance to *Aedes vexans* ecology. The species relies on temporary floodwaters for egg hatching, with breeding sites often located in areas that flood regularly. Exceptional flood events can substantially expand breeding habitats, leading to mass emergence events [25]. Yet, none of the reviewed models explicitly accounted for flooding dynamics. Moreover, long-term averaged variables commonly used in species distribution models tend to obscure such short-term fluctuations. As a result, current models likely fail to represent the full complexity of *Aedes vexans* habitats. Including flood-related variables could significantly improve model realism and ecological completeness. Nevertheless, such data are not universally accessible, and their predictive power may appear limited in models focused on adult occurrences, as most available records pertain to adult mosquitoes. The frequent use and high importance of temperature, precipitation, and land-cover variables—such as water and vegetation—in species distribution models for *Aedes vexans* align with the findings of a broader review study covering multiple mosquito species [29].

Another key finding concerns the frequent use of variables related to human settlements, particularly those in the ‘Artificial’ subcategory. While *Aedes vexans* may indeed benefit from blood-meal availability in densely populated areas, the apparent importance of these variables in many species distribution models likely results from sampling bias. Most occurrence records originate from easily accessible, urbanized areas [61,62]. Among the eight studies that included ‘Artificial’ variables, only two corrected for spatial sampling bias—and neither identified ‘artificial’ as an important predictor. In contrast, five of the six studies without bias correction reported ‘Artificial’ as highly relevant. A similar pattern was found for the related ‘Human’ subcategory, underscoring the often-addressed substantial effect of uncorrected sampling biases on model outcomes [63,64,65,66,67,68,69,70,71].

Current models also neglect the full host range of *Aedes vexans*, focusing solely on humans and livestock, despite evidence that the species feeds on a wide variety of hosts, including birds and rodents [25,72,73,74]. Incorporating variables that represent all relevant host groups could markedly improve the accuracy of species distribution models, particularly in regions where non-human hosts are abundant.

Only 2 of the 23 reviewed models explicitly specify which of the four recognized *Aedes vexans* subspecies they represent. Subspecies of *Aedes vexans* exhibit distinct geographic distributions [75], a factor rarely accounted for in current models. Consequently, it is often unclear whether the different subspecies occupy partially distinct ecological niches. To date, subspecies-specific modelling has been performed only for *Aedes vexans vexans* [41,42], and no tailored distribution models exist for the remaining subspecies, limiting our understanding of their potential differentiation.

Our review highlights a frequent mismatch between the spatial scales of the variables and those of the distribution models. While over half of the reviewed studies were conducted at national or sub-national scales, many relied on global or continental datasets such as CORINE, WORLDCLIM, MODIS, or GLOBCOVER [57,58,59,60]. Datasets covering global and continental scales are particularly valuable for large-scale analyses, as they provide key insights into historical dynamics and potential future global changes. However, conducting research on local or regional scales using global or continental datasets can obscure local ecological gradients and lead to inaccurate habitat-suitability predictions, as global or continental datasets often fail to capture fine-scale environmental heterogeneity [76,77,78]. Additionally, they are often biased [79,80], which can introduce artefacts into the distribution model for species with specific microhabitat requirements, such as *Aedes vexans*. However, various methods and recent advances exist to improve the quality of global datasets used at regional or local scales. Approaches that integrate regional or local data with global datasets appear promising for enhancing input data, even for areas lacking gridded datasets specifically compiled for that location [76,81]. Other methods focus on correcting biases in existing data products [82,83,84]. Our findings align with concerns raised by Lippi et al. [29], who noted that many mosquito species distribution models lack biological justification in variable selection and often neglect to address collinearity. This reflects a general deficit in ecological hypothesis testing, which may lead to oversimplified or misleading predictions. Our review confirms this issue for *Aedes vexans*, highlighting the need for more biologically grounded approaches in future modelling efforts.

### Limitations and Methodological Considerations

Several challenges arose during the review process, and these may have influenced the interpretation and generalisability of our findings. One key limitation was the inconsistent reporting and classification of explanatory input variables across studies. Often, variables were described vaguely or referenced only by the data product from which they were derived, without further specification. This limited the precision with which variables could be compared or interpreted.

To manage this heterogeneity, we developed a hierarchical classification scheme with major categories and subcategories. While this structure allowed us to organise and analyse a large number of variables effectively, it also introduced a trade-off: broader groupings may overgeneralise ecologically distinct variables, potentially masking differences in their relevance for *Aedes vexans* habitat.

Another major limitation was the frequent lack of reporting on variable importance. Sixty percent of the reviewed studies did not quantify or describe the contribution of individual variables to model performance. This omission creates uncertainty in evaluating which variables truly influence model predictions. Notably, Lippi et al. [29] also reported that nearly 20% of distribution-modelling studies on mosquitoes failed to report variable importance, indicating that this issue persists across the broader field, albeit to a lesser extent. Moreover, retrospective assessment of variable importance was usually not possible, as only one study partially shared its code and just 17% provided access to datasets or model outputs. Without standardised reporting practices and the open sharing of data and model results, it remains challenging to trace back model inputs. Initiatives advocating for greater transparency and reproducibility in ecological modelling represent promising steps toward improving the reliability and comparability of future studies [85].

## 5. Conclusions

Our review highlights a broader tendency in species distribution modelling toward a data-driven, convenience-based “all-in, goods-out” variable-selection approach [3,29]. While such models may yield acceptable results for generalist species with broad ecological niches and high detectability, they are poorly suited to species like *Aedes vexans*, whose distribution is driven by dynamic, temporally variable processes such as flooding. Modelling such species requires variables that are specifically adapted to these dynamic conditions and directly capture the spatial variation.

To improve model quality, a more targeted, hypothesis-driven selection of ecologically meaningful variables is essential [86]. Our classification framework, which ranks variables based on their usage frequency and reported predictive performance, offers a structured starting point for variable selection.

The grouped variables introduced in this study serve as metavariables that provide a structured approach for organizing the large set of individual input variables. These metavariables capture the main environmental and ecological dimensions relevant to the life cycle and habitat requirements of *Aedes vexans*. Their utility extends beyond the specific dataset used here: on a general level, they allow for the identification of key dimensions that should be considered when constructing species distribution models. At a more detailed level, they facilitate the selection of specific datasets that accurately capture these dimensions or help to identify gaps where relevant data are missing, such as absence of flood-frequency data from the *Aedes vexans* models. This dual applicability highlights the value of metavariables for both guiding the overall modelling strategy and informing the choice of input variables.

Beyond its application to *Aedes vexans*, this systematic approach is transferable to other species, provided that sufficient modelling literature exists. It can facilitate more transparent, reproducible, and ecologically grounded model development by identifying both consistently used variables and overlooked but potentially relevant predictors. Nevertheless, variable relevance remains inherently species- and context-specific. Our classification should therefore be adapted to the ecological traits of the target species and the objectives of each modelling study; however, variable selection in future models should not be based on prior use alone, but must also reflect the variable’s ecological plausibility as a factor limiting the spatial distribution of the species.

## Figures and Tables

**Table 1:** Categorization and description of the explanatory variables used in the 28 reviewed species distribution models of *Aedes vexans*. The table lists each variable category and subcategory, along with a brief description of their content. If labels differ between the dataset and the figures, the dataset label is shown in parentheses. The original variable names used in the reviewed studies, together with their classifications, are available in the accompanying data repository.

Category	Description	Subcategory	Description
Land characteristic	Data describing the physical features and conditions of the land, often observable from a ‘top-down’ perspective.	Artificial	Details about human-made structures and landscapes, such as buildings, roads, and urban developments.
		Bare	Details about areas with minimal or no vegetation, such as deserts, rocky regions, or barren soils.
		Infrared	Metrics derived from infrared sensing, including vegetation health, water stress, or thermal properties.
		Terrain	Data on landscape features, such as elevation, slope, or landforms.
		Vegetation	Information about plant life, such as vegetation types, density, or green cover.
		Water	Data on water bodies, including rivers, lakes, ponds, and other forms of standing or flowing water.
		Wetland	Information on marshes, swamps, bogs, or areas seasonally saturated with water.
		Missing data (-)	Missing or unavailable values (e.g., cloud cover).
		Not specified (unspec.)	Variables without detailed classification.
Population	Data on the size or distribution of populations within a given area.	Distance	Measures of distance to the nearest population center.
		Human	Data on human activities, population density, or anthropogenic impacts.
		Other animal	Information about the presence or activities of non-human animal species.
Water characteristic	Data describing the physical and chemical attributes of water bodies.	Amount	Information about the amount of water available to a system or organism.
		Quality	Data on the chemical or biological quality of water.
		Soil water	Metrics related to soil moisture or groundwater levels.
Weather	Data describing atmospheric conditions.	Temperature	Metrics related to temperature and associated variations.
		Precipitation	Data on rainfall and other forms of precipitation.
		Radiation	Details about solar radiation, influencing environmental conditions.
		Humidity	Information on atmospheric moisture levels.
		Not specified (unspec.)	Variables without detailed classification.

## Data Availability

The data presented in this study are openly available in Zenodo at https://doi.org/10.5281/zenodo.14603893.

## Supplementary Materials

The following supporting information can be downloaded at: https://www.mdpi.com/article/10.3390/insects16101061/s1, Checklist: PRISMA checklist; Checklist: PRISMA abstract checklist.
